# Progression of metabolic syndrome and associated cardiometabolic risk factors from prepuberty to puberty in children: The PUBMEP study

**DOI:** 10.3389/fendo.2022.1082684

**Published:** 2022-12-19

**Authors:** Carmela de Lamas, Anton Kalén, Augusto Anguita-Ruiz, Alexandra Pérez-Ferreirós, Rosaura Picáns-Leis, Katherine Flores, Luis A. Moreno, Gloria Bueno, Ángel Gil, Mercedes Gil-Campos, Concepción M. Aguilera, Rosaura Leis

**Affiliations:** ^1^ Unit of Investigation in Human Nutrition, Growth and Development of Galicia (GALINUT), University of Santiago de Compostela (USC), Santiago de Compostela, Spain; ^2^ Pediatric Nutrition Research Group, Institute of Sanitary Research of Santiago de Compostela (IDIS), University Clinical Hospital of Santiago - University of Santiago de Compostela (CHUS–USC), Santiago de Compostela, Spain; ^3^ Department of Biochemistry and Molecular Biology II, Institute of Nutrition and Food Technology “José Mataix”, Center of Biomedical Research, University of Granada, Armilla, Granada, Spain; ^4^ Instituto de Investigación Biosanitaria ibs, Granada, Spain; ^5^ The Center for Biomedical Research Network Physiopathology of Obesity and Nutrition (CIBEROBN), Institute of Health Carlos III (ISCIII), Madrid, Spain; ^6^ Institute for Global Health (ISGlobal), Barcelona, Spain; ^7^ Unit of Pediatric Gastroenterology, Hepatology and Nutrition, Pediatric Service, University Clinical Hospital of Santiago (CHUS), Santiago de Compostela, Spain; ^8^ Metabolism and Investigation Unit, Reina Sofia University Hospital, Maimónides Institute for Biomedical Research of Córdoba (IMIBIC), University of Córdoba, Córdoba, Spain; ^9^ GENUD (Growth, Exercise, NUtrition and Development) Research group, University of Zaragoza, Institute of Sanitary Research of Aragón (IIS Aragón), Zaragoza, Spain; ^10^ Agri-food Institute of Aragon (IA2), Zaragoza, Spain; ^11^ Unit of Pediatric Endocrinology, University Clinical Hospital Lozano Blesa, Zaragoza, Spain

**Keywords:** adolescent, cardiometabolic risk factors, child, metabolic syndrome, obesity, overweight, puberty

## Abstract

**Introduction:**

Metabolic syndrome (MetS) is a cluster of clinical and metabolic alterations related to the risk of cardiovascular diseases (CVD). Metabolic changes occurring during puberty, especially in children with overweight and obesity, can influence the risk of developing chronic diseases, especially CVD.

**Methods:**

Longitudinal study based on the follow-up until puberty of a cohort of 191 prepubertal Spanish boys and girls without congenital, chronic, or inflammatory diseases: undernutrition: or intake of any drug that could alter blood glucose, blood pressure, or lipid metabolism. The following parameters were used to determine the presence of MetS: obesity, hypertension, hyperglycemia, hypertriglyceridemia, and low HDL-c.

**Results:**

A total of 75·5% of participants stayed in the same BMI category from prepuberty to puberty, whereas 6·3% increased by at least one category. The prevalence of MetS was 9·1% (prepubertal stage) and 11·9% (pubertal stage). The risk of presenting alterations in puberty for systolic blood pressure (SBP), plasma triacylglycerols, HDL cholesterol (HDL-c), and HOMA-IR was significantly higher in those participants who had the same alterations in prepuberty. MetS prevalence in puberty was predicted by sex and levels of HOMA-IR, BMI-z, and waist circumference in the prepubertal stage, in the whole sample: in puberty, the predictors were levels of HOMA-IR, BMI-z, and diastolic blood pressure in participants with obesity. Two fast-and-frugal decision trees were built to predict the risk of MetS in puberty based on prepuberty HOMA-IR (cutoff 2·5), SBP (cutoff 106 mm of Hg), and TAG (cutoff 53 mg/dl).

**Discussion:**

Controlling obesity and cardiometabolic risk factors, especially HOMA-IR and blood pressure, in children during the prepubertal stage appears critical to preventing pubertal MetS effectively.

## Introduction

Obesity has grown steadily over the past decades in all ages (1). In 2016, the prevalence of obesity was estimated at 50 million girls and 74 million boys worldwide (1). The prevalence of excess weight (overweight and obesity) in Europe, in children aged 2 to 10 years, ranges from less than 10% in the northern regions to more than 40% in the southern countries (2). Obesity in children is associated with comorbidities during this stage of life and increases the risk of diseases during adulthood (3, 4).

The metabolic syndrome (MetS) in adults is well-defined as a cluster of cardiovascular and diabetes risk factors including abdominal obesity, dyslipidemia (alterations in plasma levels of triacylglycerols due to excess or HDL-c due to deficiency), carbohydrate metabolism dysfunction, and hypertension (5). In children otherwise, there is lack of consensus on how to define MetS and which specific cut-off points should be used for each of the altered components. In a previous work from our group by Olza et al. (2011) (6), all internationally-accepted MetS definitions were compared according to their prevalence in a cohort of Spanish children with obesity, concluding that single cut-off points cannot be used to define abnormalities in children. Instead, values above the 90^th^, 95^th^, or 97^th^ age- and sex-adjusted percentiles extracted from big representative epidemiological cohorts are recommended as cut-offs. Among suggested altered components participating in the definition of MetS, insulin resistance (IR), has been proposed to be critical, since it is one of the main signs of glucose intolerance (7). This disorder is related to organ, cellular, and biochemical alterations like increased endoplasmic reticulum oxidative stress, modified production of several hormones, augmented secretion of pro-inflammatory cytokines in adipose tissue, liver, and muscle, and elevated levels of biomarkers of endothelial vessel damage and blood coagulation homeostasis (8, 9). Nevertheless, this alteration is not considered by the majority of the currently accepted definitions, despite several claims for its inclusion have been recently raised (9).

The first adaptations of the MetS diagnostic criteria in children were carried out in the nineties of the last century (10, 11). Since then, more than 40 different definitions, including different sets of features and cut-offs, have been reported (12). The incidence of MetS in children under 18 years of age ranges from 0.2% to incidences greater than 12% (13–15). In 2015, a study determined the prevalence of MetS in the general population in Spain with the most widely used pediatric definitions (of which 5.7% employs NCEP-ATPIII criteria and 3.8% the International Diabetes Federation (IDF) criteria in adolescents between 12 and 17 years old) (16). In the group of children with obesity, the agreement between the two definitions was strong but lower in normal weight adolescents (16). In our previous from 2011, we demonstrated that an incidence that ranged between 7.6 and 30.8% during the prepubertal stage and between 9.7 and 41.2% in the pubertal stage depending on the used criteria (6). Likewise, various cross-sectional studies have reported a parallel increase in the incidence of obesity and MetS in all stages of childhood, regardless of the criteria used to define it (14, 15). Besides, MetS is directly related to obesity, and its incidence is greater as its severity increases (17, 18).

The body composition, with an increase in fat mass, and hormonal and metabolic changes occurring during puberty, such as increased IR, can influence the risk of developing chronic diseases, especially CVD (19, 20). These changes appear to affect individuals with or without overweight differentially (21). Although a clear positive correlation has been established between age and MetS in adults, contradictory results have been reported in children (14). Some studies have shown significant differences in MetS prevalence between the prepubertal and pubertal stages (14, 15, 21). However, there is scarce information about how puberty affects the progression of MetS and associated cardiometabolic risk factors in longitudinal studies.

Hence, the PUBMEP study aimed to evaluate the prevalence of MetS and the progression of cardiometabolic risk factors related to it from prepuberty to puberty in a cohort of Spanish children. In this work, we also evaluate the utility of prepubertal risk factors, such as IR, not included in many of the current accepted MetS definitions for the prediction of pubertal MetS.

## Materials and methods

### Study design

The PUBMEP is a longitudinal study based on the follow-up of a cohort of children who had previously participated in the GENOBOX study (22–29). This project studied the relationship between genetic variants, markers of oxidative stress and inflammation, lifestyle, and cardiovascular risk in 1699 children and adolescents. Inclusion criteria: Prepubertal boys and girls at the time of the GENOBOX study who have already reached puberty at the time of the PUBMEP study start, were invited to participate. The following characteristics were considered as exclusion criteria: the presence of congenital, chronic, or inflammatory diseases or undernutrition: intake of any drug that could alter blood glucose, blood pressure or lipid metabolism: not being able to comply with the study procedures and being participating or having participated in the last three months in an investigation project.

A total of 374 subjects were contacted in the PUBMEP study, of which 49 were not located, 36 could not participate because they have changed their place of residence or meet any of the exclusion criteria and 98 declined the invitation. One hundred ninety-one answered affirmatively, and their parents or legal guardians accepted an appointment to receive all the information related to the PUBMEP study. **Figure S1** depicts the flow chart of selected subjects.

### Ethical considerations

The multicentric PUBMEP study was designed following the ethical principles of human research according to the seventh revision of the declaration of Helsinki (30) and current Spanish legislation on clinical research in humans. Moreover, the study was approved by the corresponding ethic committees on each of the participating centers (Code IDs GENOBOX: Córdoba01/2017, Santiago 2011/198, Zaragoza 12/2010 and PUBMEP: Córdoba 260/3408, Santiago 2016/522, Zaragoza 22/2016, Granada 01/2017). Parents and legal guardians and children over 12 years signed an informed consent before starting their participation.

### Anthropometric parameters and pubertal stage assessment

The anthropometric evaluation was carried out with the participants in underwear or light sportswear and barefoot. Weight (kg) with an electronic medical scale (SECA^®^ 701 – model class III digital display, Germany): height (cm) with a Harpenden wall-mounted stadiometer with a high-speed counter (Holtain^®^ Ltd, United Kingdom): bicipital, tricipital, subscapular and suprailiac skin folds (SF) were determined with a skinfold Caliper (Holtain^®^, Wales, UK): and waist and hip perimeter (cm) with an inextensible tape measure (SECA^®^, Germany). All parameters were determined with standardized methods in which researchers from all participating centers were previously trained. The body mass index (BMI) [(weight (kg)/height (m)^2^] was calculated and children were classified as having normal weight, overweight or obesity, according to BMI by using the Cole et al. (31) sex and age cut-offs for children. The waist circumference (WC) was related to the Spanish percentile tables of Ferrández et al. (32). The waist-to-hip ratio (WHR) was obtained. The assessment of the pubertal stage was carried out following the Tanner classification (33) and confirmed the prepubertal stage with a hormonal study. Systolic and diastolic blood pressure (SBP and DBP, respectively) were determined twice, after five minutes of rest, on the left arm of each participant while he was sitting. The measurement was performed with digital blood pressure monitor with a suitable cuff an (mofel M3, OMRON^®^, Japan). The mean values, expressed in millimeters of mercury adjusted for sex, age and height, were classified according to international references (34).

### Biochemical analyses

The lipid profile and carbohydrate metabolism were studied in a blood sample collected after 12 h of fasting and without having performed more than 2 h of physical activity during the previous 24 h. The following parameters were determined to assess the lipid profile: total cholesterol and triacylglycerols (TAG) (Advia 2400 Chemistry system: Siemens healthcare diagnostics, Erlangen, Germany) and HDL-c and low-density lipoprotein cholesterol (LDL-c) (SAS-3 cholesterol profile kit – Helena Biosciences Europe: Tyne and Wear, UK). Plasma fasting glucose (Advia 2400 Chemistry system: Siemens healthcare diagnostics, Erlangen, Germany) and insulin (Advia centaur XP analyzer, Siemens healthcare diagnostics, Erlangen, Germany) determinations were used to calculate the homeostasis model assessment of insulin resistance (HOMA-IR) index, which allowed us to assess carbohydrate metabolism.

### Metabolic syndrome definition

To determine the presence of MetS we followed the recommendations by Olza et al. (2011) (6), which states the use of sex- and age-specific international criteria and cutoff points: obesity (BMI>95p) according to Cole et al. (2000) (31), and, at least, two altered components among the following: hypertension (BP z-score>95p) (34), carbohydrates metabolism dysfunction (hyperglycemia >100mg/dL) (35), hypertriglyceridemia (TAG z-score>95p) (36–39) and low HDL-c (HDL-c<5p) (40). When selecting the variable associated with the determination of fat mass, different options e.g., BMI, WC or WHR and cut-off points were evaluated. There was a very good correlation between all of them and BMI according to Cole et al. (2000) criteria (31) was finally selected because of its higher frequency of use. This definition therefore employs the latest available reference charts for Caucasians populations covering both prepubertal and pubertal stages.

### Statistical analysis

For the final analysis, 10 participants belonging to the normal weight group who were outliers (had extreme values > p99) in any of the variables analyzed and 38 participants in whom some data were missing were excluded. Finally, 143 participants were included in the analysis.

A complete picture of the full analytical procedure is presented in **Figure S2**. The prepubertal-to-pubertal progression of body composition and cardiometabolic risk markers were analyzed using three approaches: 1) evaluation of the overall progression and differences between girls and boys, 2) prospective analysis of participants grouped based on their BMI status and MetS prevalence in prepuberty, and 3) retrospective analysis of participants grouped by MetS prevalence in puberty. For all three approaches, mixed-effects models were fitted for each of the analyzed phenotypes to investigate between- and within-group differences. The models included fixed effects for puberty stage, group, and their interactions, to allow within and between-group comparisons: and Tanner stage and sex to correct for any differences in pubertal status and sex between groups. A random effect for participants was included to account for repeated measures. All variables, except age, Tanner stage and glucose, were log-transformed. The models were used to test for progression in analyzed variables from prepubertal to pubertal stage within each group, pairwise differences at the prepubertal stage between groups, and pairwise differences in the prepubertal-pubertal evolution between groups. All tests were calculated using the Kenward-Roger method for degrees of freedom and corrected for false discovery rate using Benjamini–Yekutieli procedure. The models did not show a problem of non-normality or heteroscedasticity of residuals.

Further, to assess the within-participant association of cardiometabolic risk in prepuberty and puberty, logistic regressions were used to test if the prevalence of MetS in prepuberty affected the risk of MetS in puberty. Likewise, logistic regressions were used to test if altered levels of DBP, SBP, TAG, HDL-c, HOMA-IR and glucose in prepuberty affected the risk of having altered levels of these variables in puberty. The purpose of including HOMA-IR in these analyses was to demonstrate the relevance of prepubertal IR for the development of future MetS (even not participating in its definition). The cut-off point of HOMA-IR was considered 2.5 in the prepubertal stage: 3.38 in pubertal boys and 3.905 in pubertal girls. These IR cutoff values were extracted according to the 95th HOMA-IR percentile in a subset of 677 prepubertal and 778 pubertal Spanish children (41, 42). Although the ideal situation would involve to extract particular percentile cut-offs for each tanner stage, this was not possible in our study given the lack of enough individuals in each category for extracting reliable percentiles. Therefore, we claim to responsible use if extending these cutoffs to other populations, especially if individuals correspond to late tanner stages.

Two statistical approaches were used to test which cardiometabolic risk markers in prepuberty can predict MetS prevalence in puberty. First, a backward stepwise logistic regression tested which prepubertal levels of BMI z-score, WC, SBP, DBP, TAG, HDL-c, LDL-c, glucose, HOMA-IR and age, together with Tanner stage in puberty, would influence the prediction of MetS in puberty. The predictors did not suffer from multicollinearity (max VIF = 1.81). This procedure was repeated for the subsample of participants that presented obesity in prepuberty.

Secondly, two fast-and-frugal decision trees were constructed so that they potentially identify prepubertal children at risk of developing MetS in puberty (43). Fast-and-frugal decision trees provide easy to interpret decision trees, which are robust to overfitting (43). They have been successfully used to describe decision processes and provide prescriptive guides for effective real-world decision-making in medicine (44, 45), amongst other areas. To identify children at risk of developing MetS, a high sensitivity (i.e., correctly classifying individuals with MetS in puberty) over a high specificity (i.e., correctly classifying individuals without MetS in puberty) was considered more important for the purpose in this study. Therefore, the trees were evaluated using a weighted accuracy metric. The trees with the best performance, when sensitivity was weighted 1.5 and 1.2 times higher than the specificity, were chosen. We used leave-one-out cross-validation (LOO-CV) to estimate how the trees were expected to perform in general when used to make predictions on data not used during the training of the model (46). This approach involves elaborating (training) the trees using all except one observation and then test the classification performance on the last observation. This procedure is repeated for each observation. The data were stratified on MetS prevalence in puberty to ensure cases of MetS in all folds. Due to the low number of MetS cases, bootstrapping was used within each fold to increase the sample size 25 times. The full CV procedure was repeated five times, giving a total of 20 estimates. We compared the predictive value of the two trees against those obtained by receiver operating characteristic (ROC) curves using prepubertal BMI-z and HOMA-IR, individually, to classify the true positive rate (TPR) against the false positive rate (FPR) of MetS in puberty. All statistical analyses were performed using the R statistical package (3.6.0 version).

## Results

Participant characteristics, anthropometry, and plasma levels of cardiometabolic risk factors associated with the MetS in prepuberty and puberty are presented in **Table 1**. There were no significant differences between boys and girls in the prepubertal stage for any variables. At puberty, girls were significantly younger (p<0.001), and had smaller WHR than boys.

**Table 1:** Participant characteristics, anthropometry and cardiometabolic risk markers grouped by pre and pubertal stage and by sex.

Variable	Total (n = 143)			Girls (n = 71)			Boys (n = 72)		
	Pre	Pub	p-value	Pre	Pub	p-value	Pre	Pub	p-value
Age (yr)	7.8 ± 1.8	14.3 ± 1.9	<0.001	7.5 ± 1.7	13.9 ± 1.9	<0.001	8.2 ± 1.8	14.7 ± 1.7	<0.001
Tanner stage	0.0 ± 0.0	4.3 ± 1.1	<0.001	0.0 ± 0.0	4.5 ± 0.9	<0.001	0.0 ± 0.0	4.0 ± 1.1	<0.001
**Anthropometry**									
BMI (kg/m^2^)	21.6 ± 4.6	26.3 ± 6.3	<0.001	21.2 ± 3.8	26.4 ± 6.3	<0.001	22.1 ± 5.2	26.2 ± 6.4	<0.001
BMI-z	1.92 ± 2.06	1.63 ± 1.74	0.183	1.59 ± 1.65	1.77 ± 1.86	0.999	2.24 ± 2.36	1.48 ± 1.62	0.027
BMI status									
NW	43 (30.1)	49 (34.3)	0.062	20 (28.2)	25 (35.2)	0.326	23 (31.9)	24 (33.3)	0.999
OW	31 (21.7)	39 (27.3)	0.169	17 (23.9)	19 (26.8)	0.999	14 (19.4)	20 (27.8)	0.619
OB	69 (48.3)	55 (38.5)	0.012	4 (47.9)	27 (38.0)	0.350	35 (48.6)	28 (38.9)	0.333
WC (cm)	72 ± 13	85 ± 16	<0.001	70 ± 10	84 ± 15	<0.001	73 ± 14	87 ± 17	<0.001
WHR	0.92 ± 0.07	0.86 ± 0.10	<0.001	0.91 ± 0.07	0.84 ± 0.11	<0.001	0.92 ± 0.07	0.89 ± 0.08	0.093
Sum SF (mm)	72 ± 33	84 ± 26	0.002	73 ± 28	88 ± 26	0.336	70 ± 38	80 ± 24	0.022
**Cardiometabolic risk markers**									
SBP (mm Hg)	104 ± 12	113 ± 14	<0.001	105 ± 10	111 ± 11	0.605	102 ± 13	116 ± 17	<0.001
DBP mm Hg)	62 ± 9	69 ± 9	<0.001	63 ± 9	69 ± 10	0.114	61 ± 9	68 ± 8	<0.001
Cholesterol (mg/dL)	169 ± 33	157 ± 29	0.002	170 ± 32	159 ± 26	0.605	167 ± 33	156 ± 31	0.011
LDL-c (mg/dL)	99 ± 28	91 ± 23	0.003	100 ± 27	91 ± 23	0.231	98 ± 29	91 ± 24	0.090
HDL-c (mg/dL)	56 ± 15	50 ± 12	<0.001	55 ± 15	50 ± 11	0.561	56 ± 14	49 ± 13	<0.001
TAG (mg/dL)	58 ± 28	75 ± 33	<0.001	63 ± 31	79 ± 37	<0.001	54 ± 24	70 ± 28	<0.001
Glucose (mg/dL)	84 ± 7	85 ± 9	0.007	83 ± 7	86 ± 8	0.002	84 ± 7	84 ± 9	0.999
Insulin (U/L)	8.0 ± 5.9	14.8 ± 9.5	<0.001	8.7 ± 6.0	16.2 ± 9.6	<0.001	7.3 ± 5.8	13.4 ± 9.2	<0.001
HOMA-IR	1.67 ± 1.32	3.15 ± 2.13	<0.001	1.79 ± 1.30	3.49 ± 2.25	<0.001	1.56 ± 1.35	2.82 ± 1.95	<0.001
**Metabolic syndrome**									
Prevalence (%)	13 (9.1)	17 (11.9)	0.239	9 (12.7)	10 (14.1)	0.999	4 (5.6)	7 (9.7)	0.702

Statistics presented; Mean ± SD; n (%). Pre-pub difference in total are controlled for sex and Tanner stage. Pre-pub differences and differences between sexes (in text) are controlled for Tanner stage. P-values are adjusted for false discovery rate using Benjamini–Yekutieli procedure.

Pre, Prebubertal stage; Pub, Pubertal stage; NW, Normal Weight; OW, Overweight; OB, Obesity; BMI-z, BMI z-score; Sum SF, Sum of skinfolds; WC, Waist circumference, WHR, Waist-hip ratio; SBP, Systolic blood pressure; DBP, Diastolic blood pressure; LDL-c, Low-density lipoprotein cholesterol; HDL-c, High-density lipoprotein cholesterol; TAG, triacylglycerols; HOMA-IR, Homeostatic Model Assessment of Insulin Resistance.

Concerning the changes between prepuberty and puberty, a significant decrease in the prevalence of obesity was observed, with no significant differences in girls or boys. A significant increase in WC in the total sample and in girls and boys, and in the sum of skinfolds (SumSF) in the total sample and boys were also found. The WHR decreased significantly in the total sample and in girls.

SBP and DBP increased significantly with puberty, this change being significant in both the total sample and in boys. However, TC, LDL-c and HDL-c decreased with puberty, changes being significant in the full sample and in boys. TAG increased significantly in all population, girls and in boys. About carbohydrate metabolism markers, glucose increased significantly in the total population and girls, while insulin and HOMA-IR increased significantly in both boys and girls. The prevalence of MetS was higher in pubertal boys and girls compared to prepuberty, but without significant differences between them.

### Progression of categories of BMI, MetS and presence of altered cardiometabolic risk markers

**Figure 1:**
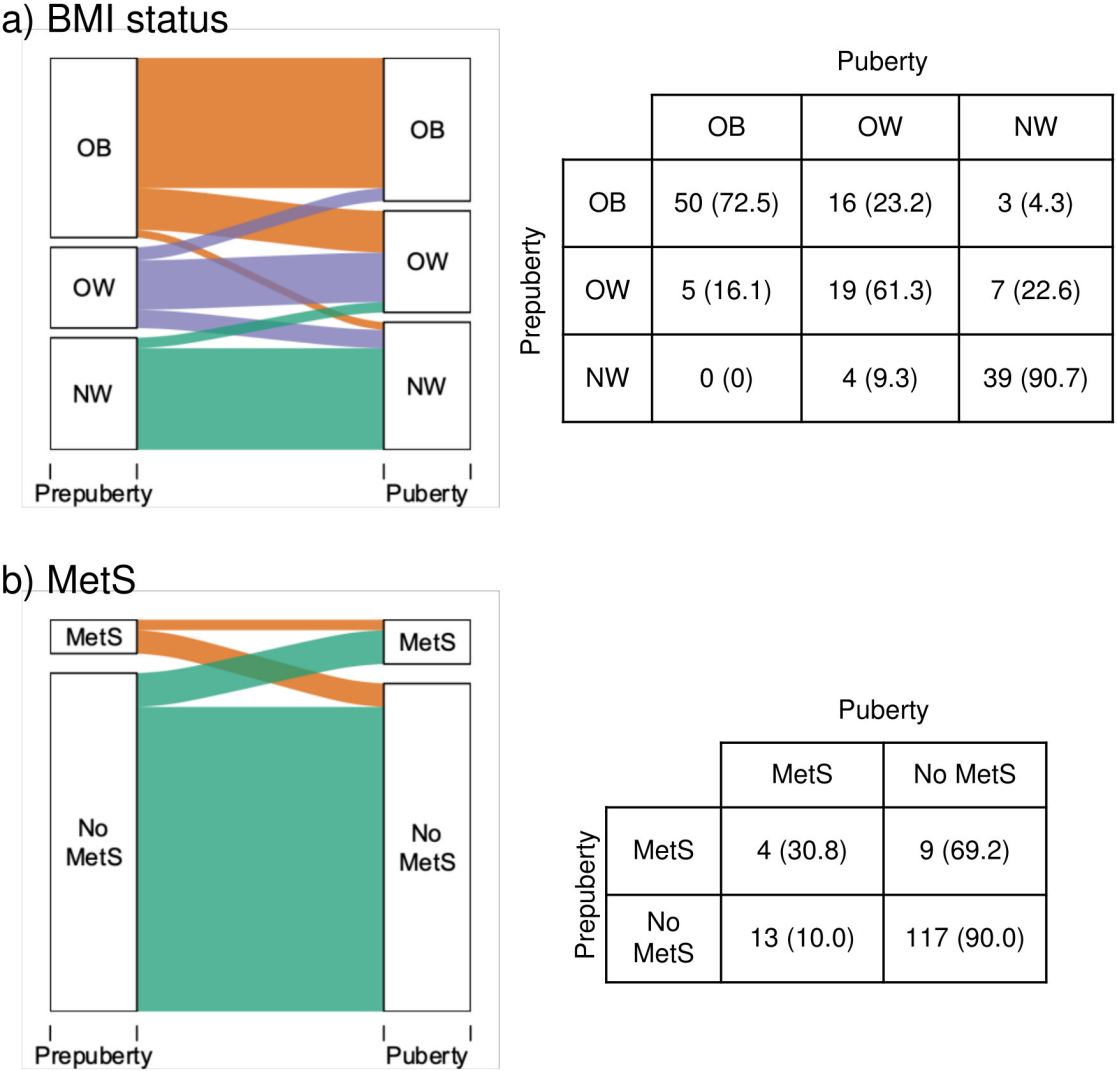
Progression from prepuberty to puberty of **(A)** BMI status and **(B)** prevalence of metabolic syndrome. Statistics presented: n (%). OB, Obesity: OW, Overweight: NW, Normal weight: MetS, Metabolic syndrome.

The progression of BMI from prepuberty to puberty can be seen in **Figure 1A**. The majority of children (75.5%) stayed in the same category of BMI from prepuberty to puberty. For example, being obese in the prepubertal stage and continue as such in the pubertal stage. On the other hand, 6.3% increased at least one category (e.g., passed from overweight to obese), and 18.2% went down at least one (e.g., from obese to overweight). Among the children who were overweight in the prepuberty 16.1% developed obesity. 72.5% of children with prepubertal obesity continued at puberty.

**Figure 2:**
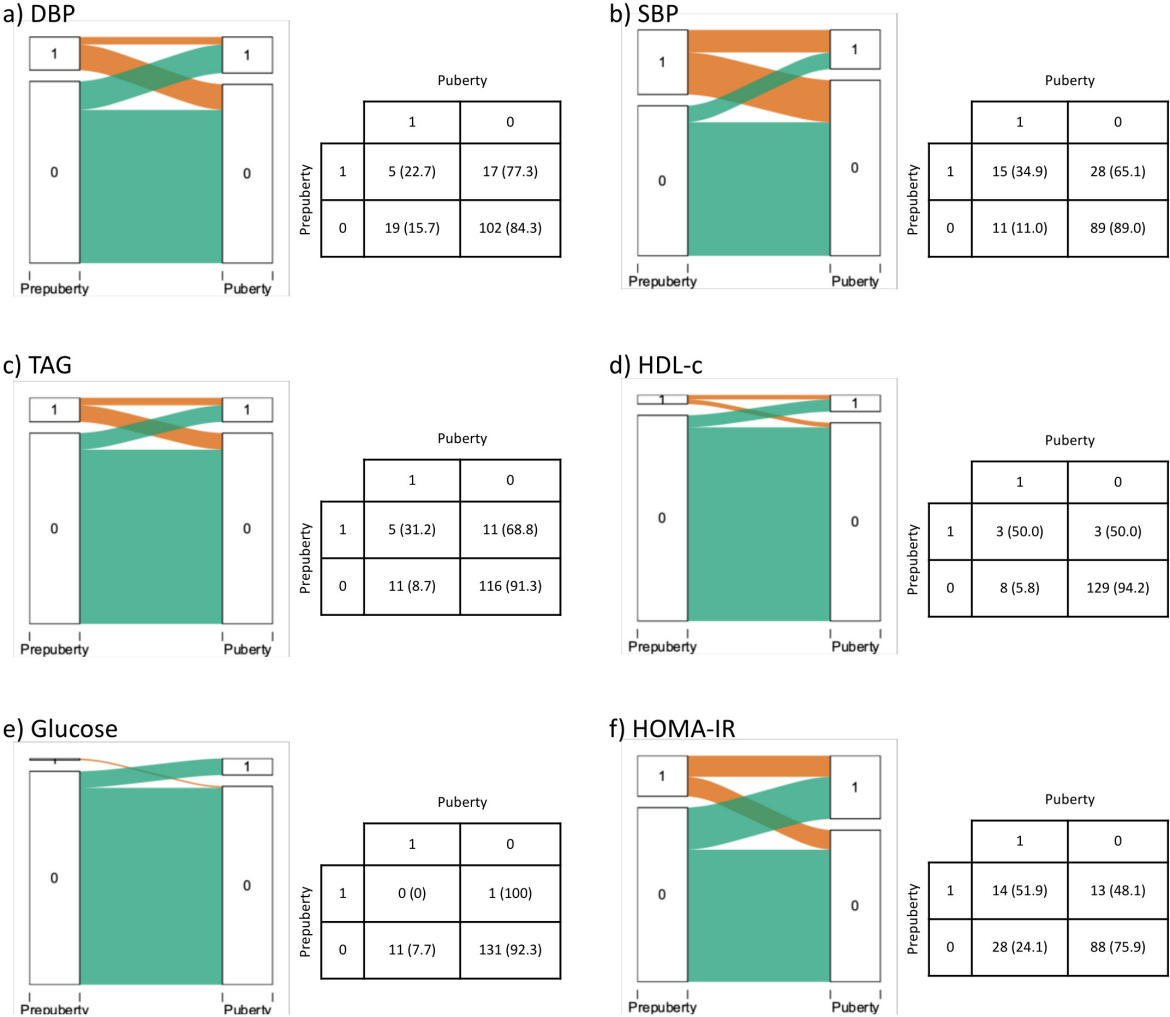
Progression of **(A)** DBP, **(B)** SBP, **(C)** TAG, **(D)** HDL-c, **(E)** Glucose and **(F)** HOMA-IR from prepuberty to puberty. 1 Indicates above the threshold, 0 below the threshold according to Olza et al. (6). Statistics presented: n (%). DBP, Diastolic blood pressure: HDL-c, High-Density Lipoprotein cholesterol: HOMA-IR, homeostasis model assessment of insulin resistance: SBP, Systolic blood pressure: TAG, Triacylglycerols.

The progression of MetS prevalence can be seen in **Figure 1B**. Participants with MetS in prepuberty had 3.08 times higher risk of having MetS in puberty (95% CI: 0.98–7.27). The progression of the individual risk factors for MetS (13): hypertension, hypertriglyceridemia, low HDL-c, hyperglycemia (fasting plasma glucose > 100 mg/dL) (47), and HOMA-IR from prepuberty to puberty are presented in **Figure 2**. High prepubertal SBP increased the risk of high pubertal SBP by 3.17 times (95% CI: 1.60–6.55). Prepubertal hypertriglyceridemia increased the risk of pubertal hypertriglyceridemia by 3.61 times (95% CI: 1.28–8.65). Low prepubertal HDL-c increased the risk of low pubertal HDL-c by 8.56 times (95% CI: 2.35–22.77). High prepubertal HOMA-IR increased the risk of high pubertal HOMA-IR by 2.15 times (95% CI: 1.27–3.44). Prepubertal high DBP and hyperglycemia were not associated with pubertal high DBP and hyperglycemia respectively.

### Prospective analysis by category of BMI and MetS prevalence in prepuberty

Participants were grouped by their prepubertal category of BMI and prevalence of MetS to analyze how obesity and MetS prevalence in prepuberty were related to the modification of anthropometry and cardiometabolic risk markers in puberty. There were no significant differences between the groups in how they progressed from prepuberty to puberty in age nor in the rest of anthropometric parameters nor in cardiometabolic risk markers. BMI-z was fairly maintained from prepuberty to puberty: however, Obesity No-MetS prepubertal children exhibited a significant decrease, being the only one to show a reduction (-0.91). In contrast, a substantial increase in WC was observed in the four considered groups. Prepubertal children with obesity, both No-MetS and MetS, had significantly higher WC than normal weight and overweight children.Moreover, a significant increase was found for the Sum SF in the normal weight group (**Table 2**).

**Table 2:** Pre and pubertal levels and change of anthropometry and cardiometabolic risk markers, grouped by BMI status in prepuberty.

Variable	Normal weight in prepuberty (n = 43)			Overweight in prepuberty (n = 31)			Obesity No-MetS in prepuberty (n = 56)			Obesity MetS in prepuberty (n = 13)		
	Pre	Pub	Δ	Pre	Pub	Δ	Pre	Pub	Δ	Pre	Pub	Δ
Age (yr)	7.9 ± 1.8	14.8 ± 1.7	6.8 ± 2.6*	8.5 ± 1.7^c^	14.6 ± 1.9	6.1 ± 2.4*	7.4 ± 1.9^b^	13.7 ± 1.9	6.3 ± 2.3*	7.7 ± 1.4	14.6 ± 1.3	6.9 ± 2.2*
Tanner stage	0.0 ± 0.0	4.3 ± 1.1	4.3 ± 1.1	0.0 ± 0.0	4.4 ± 1.0	4.4 ± 1.0	0.0 ± 0.0	4.1 ± 1.1	4.1 ± 1.1	0.0 ± 0.0	4.7 ± 0.8	4.7 ± 0.8
**Anthropometry**												
BMI (kg/m^2^)	16.2 ± 1.5	20.3 ± 2.9	4.1 ± 2.5	20.8 ± 1.9	25.3 ± 3.5	4.6 ± 3.5	25.1 ± 2.2	29.7 ± 5.3	4.6 ± 4.9	26.6 ± 4.1	34.2 ± 5.9	7.6 ± 3.7
BMI-z	-0.27 ± 0.55^bcd^	-0.10 ± 0.70	0.17 ± 0.58	1.25 ± 0.44^acd^	1.29 ± 0.98	0.04 ± 0.98	3.55 ± 1.50^ab^	2.64 ± 1.40	-0.91 ± 1.80*	3.73 ± 1.96^ab^	3.76 ± 1.43	0.03 ± 1.95
WC (cm)	58 ± 5^bcd^	71 ± 9	13 ± 8*	70 ± 8^acd^	82 ± 9	12 ± 11*	80 ± 9^ab^	94 ± 14	15 ± 13*	83 ± 12^ab^	104 ± 14	21 ± 8*
WHR	0.88 ± 0.06^c^	0.83 ± 0.12	-0.07 ± 0.09*	0.91 ± 0.08	0.84 ± 0.07	-0.07 ± 0.07*	0.95 ± 0.06^a^	0.90 ± 0.08	-0.05 ± 0.08*	0.94 ± 0.08	0.90 ± 0.07	-0.03 ± 0.04
Sum SF (mm)	35 ± 17^bcd^	83 ± 27	51 ± 33*	77 ± 22^ac^	86 ± 24	11 ± 27	94 ± 21^ab^	82 ± 26	-10 ± 37	104 ± 24^a^	93 ± 21	-7 ± 43
**Cardiometabolic risk markers**												
SBP (mm Hg)	101 ± 11^d^	107 ± 11	7 ± 15	105 ± 9^d^	112 ± 12	7 ± 11	102 ± 12^d^	117 ± 13	15 ± 15*	116 ± 6^abc^	121 ± 23	6 ± 22
DBP mm Hg)	62 ± 7^d^	67 ± 7	5 ± 9	61 ± 8^d^	68 ± 6	7 ± 8*	60 ± 7^d^	68 ± 9	8 ± 11*	76 ± 12^abc^	76 ± 17	0 ± 23
Cholesterol (mg/dL)	173 ± 31	162 ± 31	-10 ± 24	175 ± 37	154 ± 33	-21 ± 26*	162 ± 32	156 ± 24	-7 ± 26	170 ± 29	159 ± 30	-11 ± 25
LDL-c (mg/dL)	97 ± 27	90 ± 25	-8 ± 18	104 ± 33	89 ± 26	-14 ± 20*	96 ± 27	91 ± 21	-6 ± 21	108 ± 27	97 ± 25	-11 ± 20
HDL-c (mg/dL)	64 ± 15^bcd^	58 ± 14	-6 ± 12	57 ± 16^ad^	47 ± 11	-10 ± 14*	52 ± 10^ad^	47 ± 10	-5 ± 10*	42 ± 10^abc^	41 ± 9	-1 ± 8
TAG (mg/dL)	46 ± 14^bd^	67 ± 29	21 ± 28*	67 ± 35^ad^	73 ± 31	7 ± 30	55 ± 23^d^	75 ± 30	19 ± 28*	92 ± 36^abc^	101 ± 47	9 ± 46
Glucose (mg/dL)	84 ± 7	86 ± 9	2 ± 10	86 ± 6	85 ± 7	-1 ± 10	81 ± 7	84 ± 9	3 ± 11	86 ± 8	85 ± 9	-1 ± 12
Insulin (U/L)	4.6 ± 2.5^bcd^	10.2 ± 4.8	5.6 ± 5.4*	8.7 ± 5.8^a^	12.6 ± 5.8	4.0 ± 7.5*	9.3 ± 6.5^a^	18.1 ± 11.4	8.8 ± 10.5*	12.2 ± 6.9^a^	21.0 ± 10.7	8.8 ± 13.8*
HOMA-IR	0.95 ± 0.52^bcd^	2.20 ± 1.15	1.25 ± 1.26*	1.87 ± 1.36^a^	2.69 ± 1.34	0.82 ± 1.70*	1.89 ± 1.39^a^	3.84 ± 2.59	1.95 ± 2.33*	2.68 ± 1.71^a^	4.46 ± 2.40	1.78 ± 3.24*

Statistics presented: Mean ± SD: n (%). Pre-pub evolution, group differences in prepuberty and in evolution (in text) were controlled for sex and Tanner stage. P-values are adjusted for false discovery rate using Benjamini–Yekutieli procedure.

* Significant change from pre to pubertal stage within group: ^a^significant difference from Normal Weight: ^b^significant difference from Overweight: ^c^significant difference from Obesity No-MetS: ^d^significant difference from Obesity MetS.

MetS, Metabolic Syndrome; BMI-z, BMI Z-score; Sum SF, Sum of skinfolds; WC, Waist circumference, WHR, Waist-hip ratio; SBP, Systolic blood pressure; DBP, Diastolic blood pressure; LDL-c, Low-density lipoprotein; HDL-c, High-density lipoprotein; TAG, triacylglycerol; HOMA-IR, Homeostatic Model Assessment of Insulin Resistance.

In relation to cardiometabolic risk markers, SBP and DBP increased significantly at puberty in Obesity No-MetS-children (p<0.001), while in the overweight group, only a significant increase in DBP was observed (p=0.033). Obesity MetS group in prepuberty had significantly higher SBP and DBP than the rest of groups. About the lipid profile, children with Obesity MetS in prepuberty had significantly lower levels of HDL-c and higher TAG than children of all other groups. Insulin and HOMA-IR levels increased significantly with puberty in all groups: they were significantly higher in overweight and obesity, both No-MetS and MetS, groups than normal weight group (**Table 2**).

### Retrospective analysis by MetS prevalence in puberty

Participants were stratified in two groups, based on presence of MetS or no in puberty, to analyze the progression of anthropometric parameters and cardiometabolic risk markers from prepuberty to puberty (**Table 3**). The progression from prepuberty to puberty was significantly different between No MetS and MetS group for WHR, Sum SF and DBP.

**Table 3:** Pre and pubertal levels and change of anthropometry and cardiometabolic risk markers grouped by the presence or absence of metabolic syndrome in puberty.

Variable	No MetS in Puberty (n = 126)			MetS in Puberty (n = 17)			Pre diff
	Pre	Pub	Δ	Pre	Pub	Δ	p-value
Age (yr)	7.8 ± 1.8	14.4 ± 1.9	6.6 ± 2.4*	7.9 ± 1.7	13.8 ± 1.7	5.9 ± 2.3*	0.999
Tanner stage	0.0 ± 0.0	4.2 ± 1.1	4.2 ± 1.1*	0.0 ± 0.0	4.5 ± 0.9	4.5 ± 0.9*	0.999
**Anthropometry**							
BMI (kg/m^2^)	21.0 ± 4.4	25.1 ± 5.4	4.2 ± 3.6*	26.5 ± 3.2	35.0 ± 6.3	8.5 ± 4.5*	<0.001
BMI-z	1.68 ± 1.96	1.29 ± 1.47	-0.39 ± 1.41	3.71 ± 1.88	4.11 ± 1.64	0.40 ± 1.67	<0.001
WC (cm)	70 ± 12	82 ± 14	12 ± 11*	83 ± 10	108 ± 14	25 ± 10*	<0.001
WHR	0.91 ± 0.07	0.86 ± 0.10	-0.07 ± 0.08*	0.93 ± 0.07	0.93 ± 0.08	0.00 ± 0.07	0.999
Sum SF (mm)	66 ± 31	83 ± 24	20 ± 39*	108 ± 21	91 ± 35	-17 ± 51	<0.001
**Cardiometabolic risk markers**							
SBP (mm Hg)	103 ± 12	111 ± 11	9 ± 14*	109 ± 9	128 ± 23	20 ± 23*	0.314
DBP mm Hg)	62 ± 8	67 ± 7	5 ± 10*	64 ± 12	82 ± 11	18 ± 13*	0.999
Cholesterol (mg/dL)	169 ± 34	157 ± 30	-12 ± 26*	166 ± 28	159 ± 21	-7 ± 20	0.999
LDL-c (mg/dL)	99 ± 28	90 ± 24	-9 ± 20*	100 ± 30	94 ± 21	-6 ± 18	0.999
HDL-c (mg/dL)	57 ± 14	51 ± 12	-6 ± 12*	48 ± 12	40 ± 9	-8 ± 9	0.036
TAG (mg/dL)	55 ± 26	70 ± 28	15 ± 30*	80 ± 32	109 ± 47	29 ± 33*	0.004
Glucose (mg/dL)	83 ± 7	84 ± 8	1 ± 10	86 ± 7	89 ± 10	3 ± 15	0.908
Insulin (U/L)	7.1 ± 5.0	13.1 ± 7.6	6.0 ± 8.2*	14.5 ± 8.2	27.5 ± 12.4	13.0 ± 12.7*	<0.001
HOMA-IR	1.47 ± 1.09	2.76 ± 1.68	1.29 ± 1.83*	3.14 ± 1.91	6.05 ± 2.80	2.91 ± 3.04*	<0.001

Statistics presented: Mean ± SD: n (%). Pre-pub evolution, group differences in prepuberty and in evolution (in text) were controlled for sex and Tanner stage. P-values are adjusted for false discovery rate using Benjamini–Yekutieli procedure.

* Significant change from pre to pubertal stage within group.

MetS, Metabolic Syndrome; Pre diff, differences in prepuberty between the groups; BMI-z, BMI Z-score; Sum SF, Sum of skinfolds; WC, Waist circumference, WHR, Waist-hip ratio; SBP, Systolic blood pressure; DBP, Diastolic blood pressure; LDL-c, Low-density lipoprotein; HDL-c, High-density lipoprotein; TAG, triacylglycerol; HOMA-IR, Homeostatic Model Assessment of Insulin Resistance.

A significant increase in BMI, WC and sum SF and a decrease in WHR with respect to prepubertal age were observed in the No-MetS group at puberty, while in the MetS group, there was a significant increase of BMI and WC. MetS group presented significantly higher BMI, BMI-z, WC, and sum SF than No-MetS group in prepuberty.

Concerning cardiometabolic risk factors, in the No-MetS group, a significant increase in SBP and DBP, insulin and HOMA-IR were observed. On the contrary, we found a significant decrease in the lipid profile parameters (cholesterol, LDL-c, and HDL-c), except for TAG, which increased significantly. In the MetS group, a significant increase in SBP and DBP, and TAG, insulin and HOMA-IR was noticed. In prepuberty, the MetS group at puberty presented significantly higher levels of TAG, insulin, and HOMA-IR and lower levels of HDL-c. The progression of BMI-z, WC, blood pressure, TAG, HDL-c, glucose, and HOMA-IR for participants with and without MetS in puberty are presented graphically in **Figure S3**.

### Prediction of MetS prevalence in puberty

A backward stepwise logistic regression was used to ascertain which of BMI z-score, WC, SBP, DBP, TAG, HDL-c, LDL-c, glucose, HOMA-IR, and age in prepuberty was useful for the prediction of MetS in puberty. Prepubertal levels of HOMA-IR (OR=1.92: CI:1.29-2.98), BMI-z (OR=1.53: CI:1.06-2.30), WC (OR=1.09: CI:1.01-1.19), and sex (OR=5.69: CI:1.28-32.32) significantly predicted MetS prevalence in puberty (**Table S1**).

As all participants who presented MetS in puberty were obese at prepuberty, we used another stepwise logistic regression to test which cardiometabolic risk factors help to predict MetS within the group of 69 children with obesity in prepuberty. Within participants with obesity at prepuberty, HOMA-IR (OR=1.88: CI:1.19-3.28), BMI-z (OR=1.03: CI:1.00-1.05) and DBP (OR=0.92: CI:0.83-0.99) significantly predicted MetS prevalence in puberty (**Table S2**).

**Figure 3:**
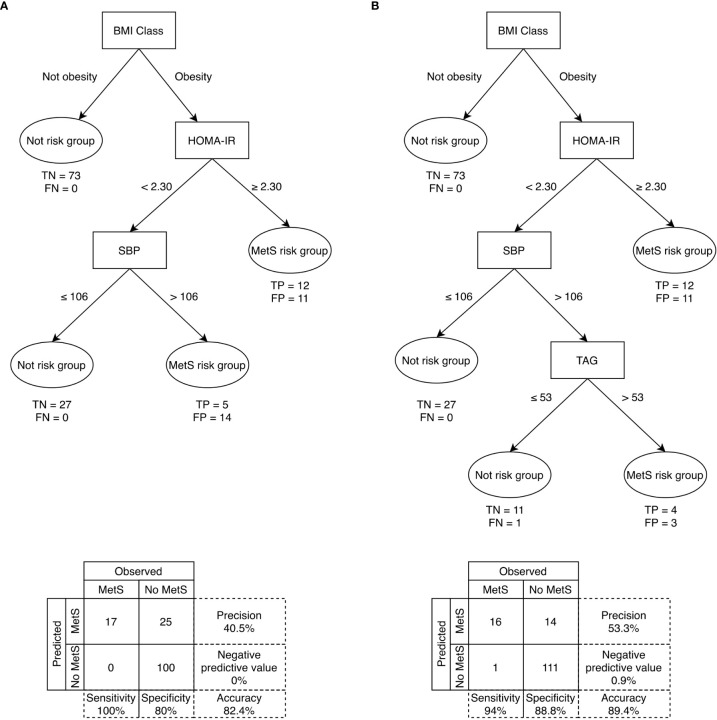
Fast-and-frugal decision trees for identifying prepubertal children in risk of developing MetS in puberty with sensitivity **(A)** 1.5 times and **(B)** 1.2 times more important than specificity. DBP= Diastolic blood pressure; FN, False negative; FP, False positive; HDL-c, High-Density Lipoprotein cholesterol; HOMA-IR= homeostasis model assessment of insulin resistance; MetS= Metabolic syndrome; SBP, Systolic blood pressure; TAG, Triacylglycerols; TN, True negative; TP, True positive. Sensitivity (i.e., correctly classifying individuals with MetS in puberty); specificity (i.e., correctly classifying individuals without MetS in puberty).

Fast-and-frugal decision trees were built to predict the risk of MetS in puberty based on body composition and cardiometabolic risk factors in prepuberty (**Figure 3**). Two trees are proposed with HOMA-IR cut-off 2,5, SBP cut-off 106 mm of Hg and TAG cut-off 53 mg/dl. One with the best accuracy is given when sensitivity is 1.5 times higher than the specificity (**Figure 3A**). The second one represents an alternate model when sensitivity is 1.2 times higher than the specificity (**Figure 3B**). The first decision tree correctly classified 82.4% of the total children and correctly classified 100% of the children with MetS in puberty. Moreover, out of the 25 false positives, 20 had at least one altered component of MetS or obesity, and five had neither altered features nor obesity. The second decision tree correctly classified 89.4% of the total children and correctly classified 94.1% of the children with MetS in puberty. Of the 14 false positives, 11 had at least one altered component of MetS or obesity, and three had neither altered features nor obesity. LOO-CV showed that the first tree had an out-of-sample sensitivity of 100%, specificity of 85.6%, and accuracy of 87.3%. The second tree showed an out-of-sample sensitivity of 94.1%, specificity of 88.8%, and accuracy of 89.4%. The classification performance of the decision trees compared with the predictive values given by ROC curves when using only prepubertal BMI-z (area under curve =0.792) or HOMA-IR (area under curve =0.786) to predict pubertal MetS, is shown in **Figure S4**. Both prediction trees exhibited higher performance than using single predictors i.e., BMI-z or HOMA-IR.

## Discussion

The hypothesis that the metabolic changes occurring during puberty is usually associated with anthropometric changes and IR leading to greater cardiovascular risk has been evidenced in the present study. Changes in anthropometric parameters related to increased cardiovascular risk were observed from prepuberty to puberty, especially WC, and in the MetS associated risk factors. A high percentage of prepubertal children maintained their BMI-z at puberty. In prepubertal children with obesity, a HOMA index >2.3 and a SBP >106 mm of Hg would identify 100% of prepubertal children with risk of MetS at pubertal age, with an accuracy of 82.4%. Indeed, the built-up decision- tree models for the prediction of MetS allowed us to determine, using only the BMI z-score, the HOMA-IR and the SBP in prepuberty, the individuals at high risk of presenting MetS at the pubertal stage. The possibility of early prediction of MetS in adolescents using simple cut-offs for cardiometabolic risk parameters at prepuberty, constitutes the major singularity and uniqueness of the present study, which may have important clinical implications in the early diagnosis and treatment of children at risk of developing premature CVD.

In our study, when evaluating the progression of BMI category, most of the participants remained in the same category (90.7% in normal weight, 61.3% in overweight and 72.5% in obesity). These results coincide with those of a narrative review published by Caprio et al. in 2020, with data collected from several studies, who estimated that approximately 80% of children with obesity maintained until adults (48).

The risk of MetS in puberty was significantly higher in participants with prepubertal MetS, what agrees with other publications (5). These results also support the data obtained from 285 participants in the Bogalusa Heart study followed for 15 years, where a positive correlation between prepubertal MetS and MetS in early adulthood was observed (49).

In the present work, within the participants who did not have prepubertal MetS, only 10% developed it in puberty. It is important to note that this 10% corresponded to individuals who, even without MetS, presented obesity in prepuberty. These results would reinforce those of other studies that conclude that individuals with obesity have a higher prevalence of MetS (14, 49). Furthermore, the incidence of MetS increases simultaneously as the BMI rises, as demonstrated by Kuschnir et al. (50).

In our study, the risk of presenting in puberty altered levels of SBP, TAG, HDL-c and HOMA-IR was significantly higher in those participants who had altered levels in prepuberty. However, this was not the case for DBP and glucose. These results are similar to those of the Québec family study, published in 2001, in which the progression of Sum SF of the trunk, mean arterial BP, HDL-c and TAG in 147 boys and girls from childhood to young adult were evaluated. Similarly, other authors shown the highest correlations for the Sum SF and HDL-c and the lowest for glucose (51).

When considering the progression of cardiometabolic risk factors by groups based on BMI and the presence of MetS in prepuberty, we observed that insulin, HOMA-IR and WC increased significantly within the four considered groups, as also reported by other authors (52). Our results indicate that prepubertal HOMA-IR, BMI-z, WC and female sex influence the prevalence of MetS. In prepubertal participants with obesity, HOMA-IR, BMI-z and SBP allowed predicting the prevalence of MetS in puberty. Many articles, both in children and adults, have pointed to BMI or WC as one of the most important predictive factors for the development of MetS (53–55). Likewise, some studies have also indicated alterations in IR and SBP as important risk factors, especially in populations at risk, such as relatives of individuals with diabetes mellitus (52, 56). Concerning the female sex, these data seem to contradict other publications that describe a greater risk of presenting MetS in boys (57) or do not describe differences between the sexes (58). The reason could be that the girls in our study had a significantly higher Tanner stage than boys, and this could be associated with a higher risk of MetS (14).

In our cohort, the HOMA-IR in the entire sample and in the prepubertal group with obesity, both in prospective and retrospective analysis, appeared the most important predictor of MetS in puberty. Most definitions of MetS in childhood do not include insulin or resistance as a feature (12), perhaps due to the difficulty of its determination and standardization of the analytical methodology among different clinical centers. However, as IR is an apparent prognostic factor of MetS in childhood, it could be interesting to use it already in prepuberty, as others have also suggested (51). Our own research group proposed it in a publication in 2015 (35). In this regard, in a recent survey, it has been shown that high BMI is not associated consistently with dyslipidemia and disturbed glucose metabolism in children and adolescents with classes III and IV obesity: therefore, measurements of cardiovascular risk factors instead of BMI seem preferable to counsel different treatment approaches (52).

Some studies (52) have shown that the measurement of adiposity but not its distribution, such as BMI, can leave out some people at risk, including children. However, when studying the predictive factors of MetS, BMI z-score or WC z-score is normally used, obtaining similar results with high concordance. After carrying out the cross-validation of the tree predictive models, it is important to emphasize that the use of these models seems to be more convenient than using only HOMA-IR or DBP individually.

## Limitations and strengths of the study

The main limitations of our study are, on the one hand, the low number of participants and the different grades of pubertal stages included. On the other hand, there is a certain risk of selection bias, since part of the participating individuals -those with the worst progression-, were followed up in the consultations of the participating hospitals, so it could have been easier to contact them or encourage them to participate in the study as they maintained contact with the research team. Another possible source of bias would be the difference in time elapsed between the two measurements (prepubertal and pubertal times) between the different participants.

The main strength of our study is the high significance obtained in the regression curves, although the number of participants was not high, as well as the high sensitivity, specificity, and accuracy of the MetS predictive models. Likewise, the fact that it was a prospective study allowed us to establish a correct temporal sequence and control how to measure the effects. Besides, standardized methodology was applied in all participated centers.

## Conclusions

A large number of children with obesity in the Spanish population already presents MetS from a very early age (prepubertal period). The majority of the children presenting MetS in early-childhood continue as such when they enter into puberty, which drastically increase their probabilities of being adults with obesity, diabetes and CVDs. Prepubertal HOMA-IR and blood pressure are again evidenced as key risk factors for the prediction of pubertal MetS, as evidenced in our machine learning-based models. Therefore, the MetS criteria not considering IR should be revisited. This paper reinforces the importance of earl-life monitoring of metabolic health in children with obesity and the initiation of preventive programs from the prepubertal stages to avoid future comorbidities in adult populations.

## Data availability statement

The original contributions presented in the study are included in the article/**Supplementary Material**. Further inquiries can be directed to the corresponding author.

## Ethics statement

The studies involving human participants were reviewed and approved by Ethic committees on each of the participating centers (Code IDs GENOBOX: Córdoba01/2017, Santiago 2011/198, Zaragoza 12/2010 and PUBMEP: Córdoba 260/3408, Santiago 2016/522, Zaragoza 22/2016, Granada 01/2017). Parents and legal guardians and children over 12 years signed an informed consent before starting their participation. Written informed consent to participate in this study was provided by the participants’ legal guardian/next of kin.

## Author contributions

Design research: CMA, RL, MG-C, GB, LM and AG. Data analysis: AK, AA-R, AP-F, CL, AG, and RL. Writing of the original draft: CL, AK, AA-R, RP-L, KF and RL. Supervision: RL, CMA and AG. Paper Editing and revision: All authors have read and agreed to the final version of the manuscript.
