# Supplementary material for: Progression of metabolic syndrome and associated cardiometabolic risk factors from prepuberty to puberty in children: The PUBMEP study
Source: Front Endocrinol (Lausanne). 2022 Dec 19;13:1082684. doi: 10.3389/fendo.2022.1082684 (PMC9806164; doi:10.3389/fendo.2022.1082684)
Supplement: Supplementary file 1 [file DataSheet_1.pdf]

Supplementary Material

Progression of metabolic syndrome and associated cardiometabolic risk factors from prepuberty to puberty in children: The PUBMEP study

Carmela de Lamas [1,2,], Anton Kalén [1,2,3], Augusto Anguita-Ruiz[4,5,6], Alexandra Pérez-Ferreirós [1,2], Rosaura Picáns-Leis[1,2,7], Katherine Flores [6,8], Luis A. Moreno[6,9,10], Gloria Bueno [6,9,10,11] , Ángel Gil [4,5,6,] Mercedes Gil-Campos[6,8], Concepción M. Aguilera [4,5,6,] *, Rosaura Leis [1,2,6,7]

Corresponding Author:

Concepción M. Aguilera

Telephone +34 626552847. E-mail: caguiler@ugr.es

1 Supplementary Figures and Tables

Supplementary Table S1. Logistic model for predicting prevalence of metabolic syndrome in puberty from the full sample.

Variable	OR	95% CI	p-value
HOMA-IR Prepubertal	1.92	1.29 – 2.98	0.002
BMI-z Prepubertal	1.53	1.06 – 2.30	0.026
WC Prepubertal	1.09	1.01 – 1.19	0.031
Sex: Girls[a]	5.69	1.28 – 32.32	0.032
DBP Prepubertal	0.94	0.86 – 1.01	0.089

[a]Boys was reference level for sex.

CI = Confidence interval; BMI-Z = BMI Z-score; DBP = Diastolic blood pressure; HOMA-IR = Homeostatic Model Assessment of Insulin Resistance; WC = Waist circumference.

Backwards stepwise model selection based on AIC was used to select the final model that best predict MetS in puberty. Variables included in the initial model were prepubertal levels of BMI-z, TAG, HDL-c, LDL-c, SBP, DBP, glucose, HOMA-IR, age, and waist circumference, as well as sex and Tanner stage in puberty.

Supplementary Table S2. Logistic model for predicting prevalence of metabolic syndrome in puberty for participants with obesity in prepuberty.

Variable	OR	OR 95% CI	p-value
HOMA-IR Prepubertal	1.88	1.19 – 3.28	0.012
BMI-Z Prepubertal	1.03	1.00 – 1.05	0.028
DBP Prepubertal	0.92	0.83 – 0.99	0.044
TAG Prepubertal	1.06	0.99 – 1.14	0.108

CI = Confidence interval; BMI-Z = BMI Z-score; TAG = triacylglycerol; DBP = Diastolic blood pressure; HOMA-IR = Homeostatic Model Assessment of Insulin Resistance.

Backwards stepwise model selection based on AIC was used to select the final model that best predict MetS in puberty. Variables included in the initial model were prepubertal levels of BMI-z, TAG, HDL-c, LDL-c, SBP, DBP, glucose, HOMA-IR, age, and waist circumference, as well as sex and Tanner stage in puberty.

Supplementary Figure S1. Flow chart of selected subjects.

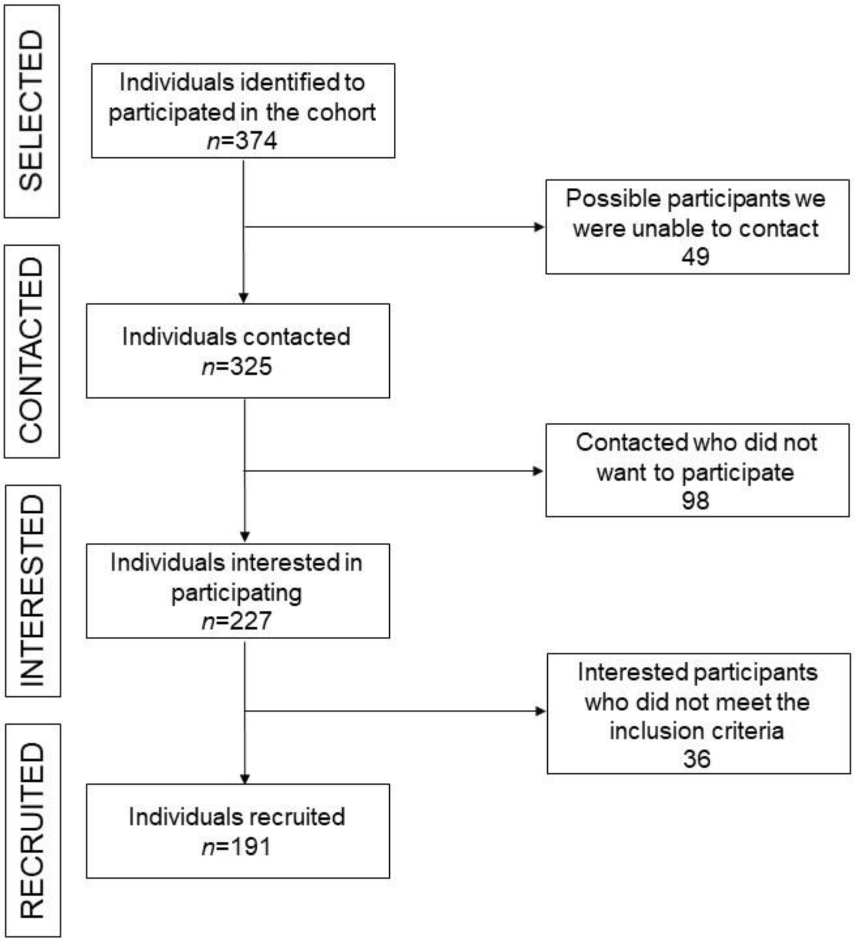

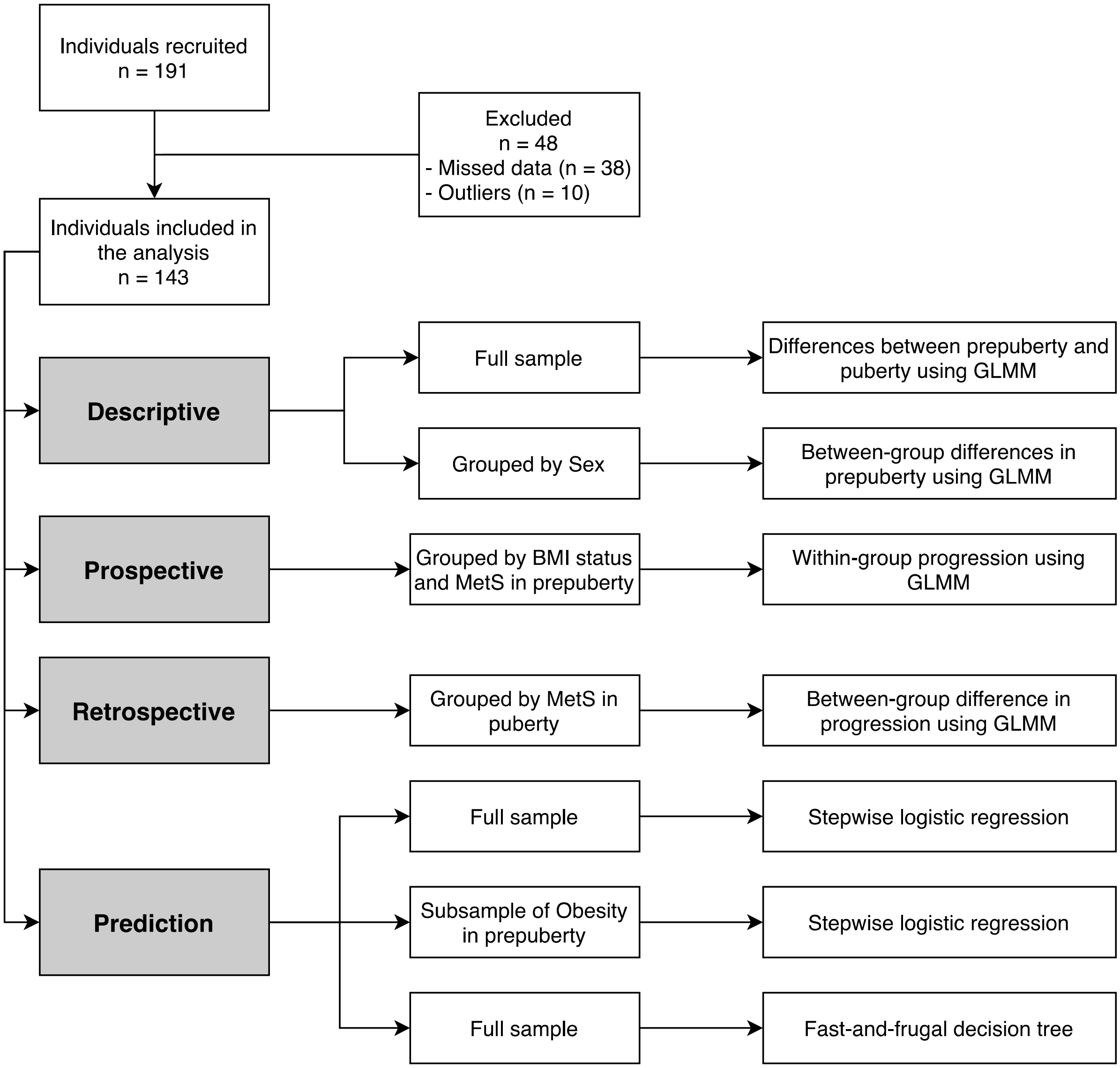

Supplementary Figure S2. Flowchart of statistical analyses. MetS= Metabolic syndrome; GLMM = generalized linear mixed model

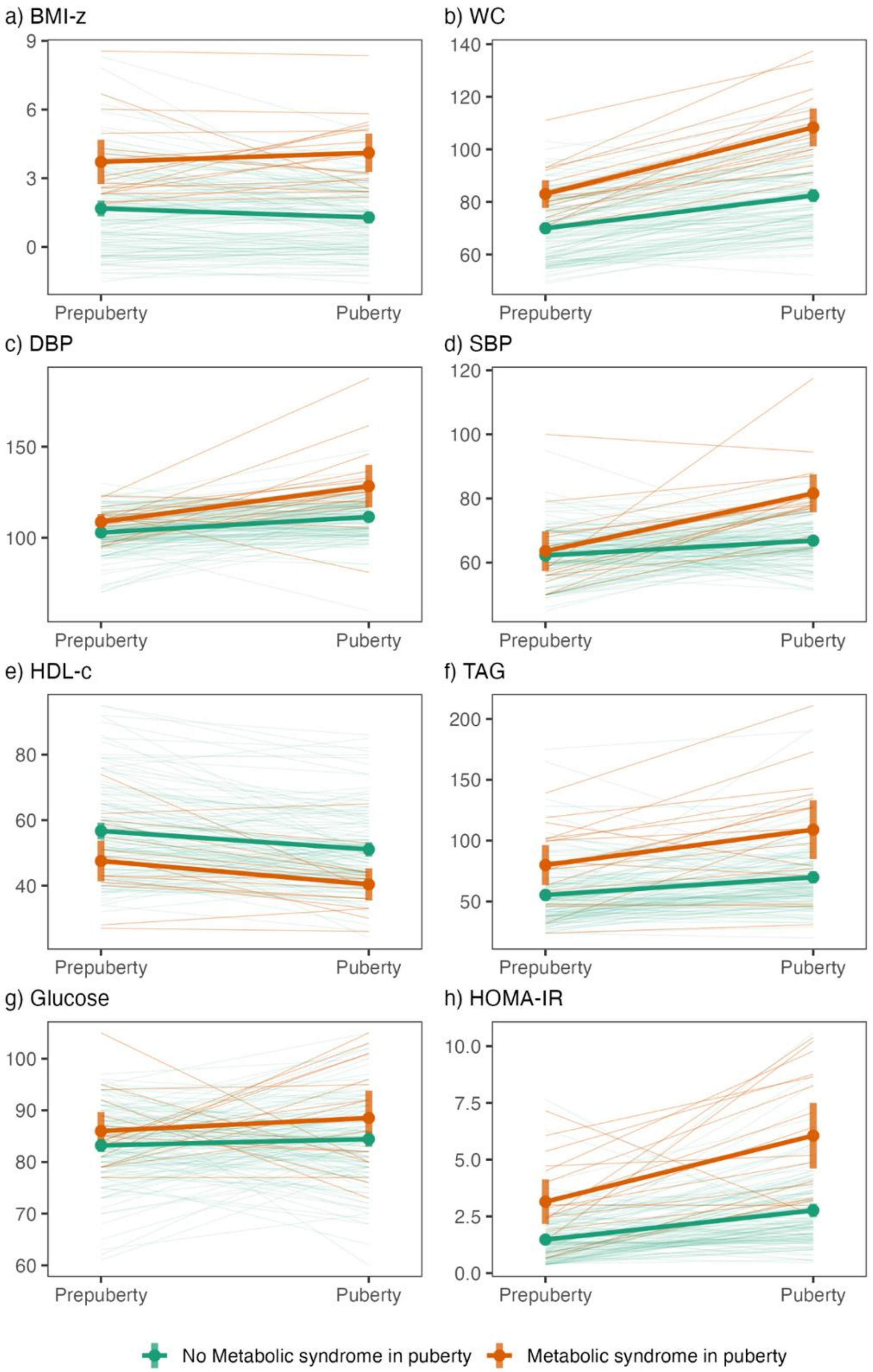

Supplementary Figure S3. Progression of a) BMI z-score, b) Waist Circumference, c) DBP, d) SBP, e) HDL-c, f) TAG, g) Glucose, and h) HOMA-IR by prevalence of MetS in puberty. Individual evolutions are presented together with mean and 95% confidence interval. DBP = Diastolic blood pressure; HDL-c= High-Density Lipoprotein cholesterol; HOMA-IR= homeostasis model assessment of insulin resistance; SBP= Systolic blood pressure; TAG= Triacylglycerols.

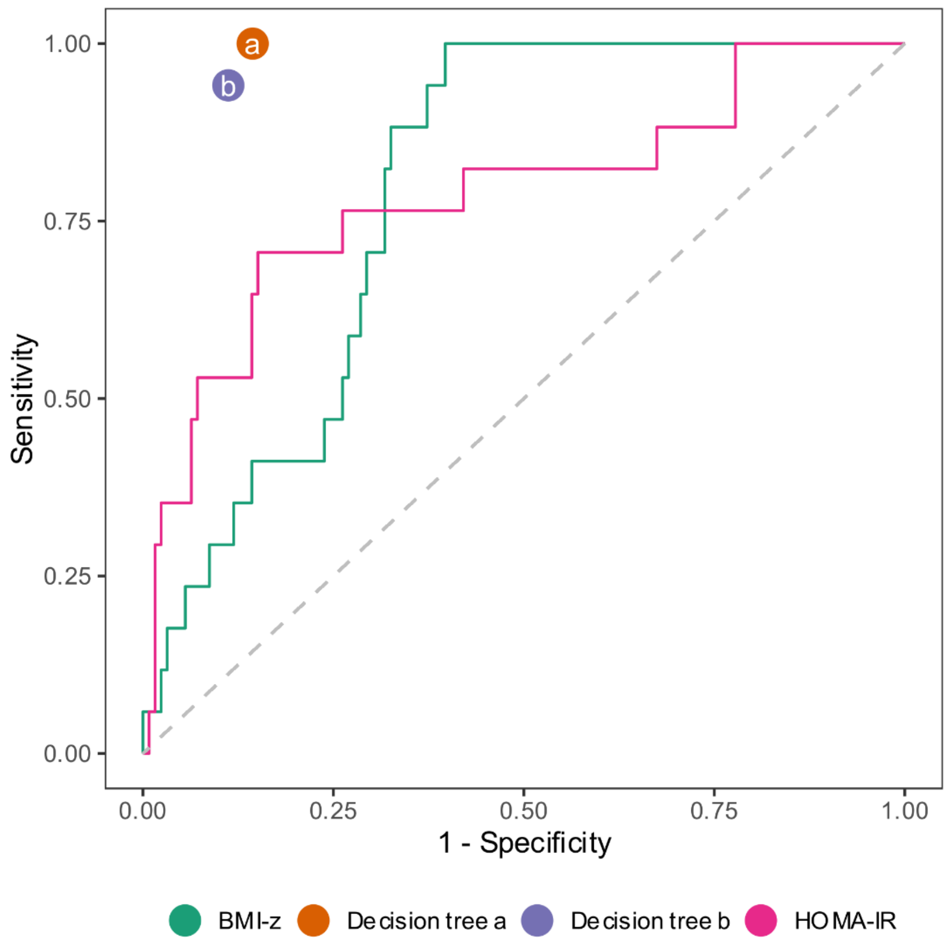

Supplementary Figure S4. Classification performance of the fast-and-frugal decision trees, compared to the ROC-curve of prepubertal BMI-z and HOMA-IR for predicting MetS in puberty. ROC= Receiver Operating Characteristics; HOMA-IR= homeostasis model assessment of insulin resistance; MetS= Metabolic syndrome.
